# Impact of a urological medical student interest group on education and career development

**DOI:** 10.1016/j.clinsp.2025.100799

**Published:** 2025-10-11

**Authors:** Eduardo Zinoni Silva Pato, Luiz Carlos Neves de Oliveira, Jose de Bessa, Gabriel Kayano Freudenthal, Guilherme Pimenta Roncete, Luiza Rafih Abud, Victor Hondo Silva de Moraes, William Carlos Nahas, Cristiano Mendes Gomes

**Affiliations:** a Division of Urology, Faculdade de Medicina da Universidade de São Paulo, São Paulo, SP, Brazil; b Department of Surgery, Universidade Estadual de Feira de Santana, Feira de Santana BA, Brazil; c Faculdade de Medicina da Universidade de São Paulo, São Paulo, SP, Brazil

**Keywords:** Urology, Medical education, Medical school, Brazil, Residency Medical

## Abstract

**Purpose:**

Medical education often provides limited exposure to urology, despite the growing prevalence of urological conditions. To address this gap, the Urological Medical Student Interest Group (UMSIG) was established in 2006. This study aimed to evaluate the educational and professional impact of UMSIG participation among its current and former members.

**Materials and methods:**

An electronic survey was distributed to 226 former and current UMSIG members between December 2023 and February 2024, collecting data on demographics, motivations for joining, perceptions of UMSIG activities, and influence on career decisions. Quantitative data were analyzed using descriptive statistics.

**Results:**

We received 111 responses (49.1 % response rate). The primary motivations for joining UMSIG included a desire to deepen urology knowledge (77.5 %) and recommendations from peers (56.8 %). Most respondents (71.2 %) reported that UMSIG enhanced their urology education beyond the standard curriculum through practical experiences. Participation also had impact on career trajectories: 52.0 % of non-urologists indicated that UMSIG influenced their current specialty, while 78.6 % of urologists cited it as a decisive factor in their choice of specialization. Overall satisfaction was high, with a median rating of 9/10, and 55.9 % considered UMSIG superior to other medical student interest groups.

**Conclusion:**

Participation in UMSIG provided a valuable supplement to formal medical education by offering practical clinical experience and research opportunities. The program influenced participants' career decisions, reinforcing interest in urology and related surgical fields. These findings underscore the important role of structured extracurricular programs in medical education, particularly in specialties with limited curricular presence.

## Highlights

• Urology is underrepresented in medical training worldwide.
• The group supported early mentorship and educational growth.
• Career choices were positively influenced by participation.
• Most participants reported high satisfaction with the program.

## Introduction

Medical education faces significant challenges due to the vast and continuously expanding body of medical knowledge. To accommodate this growing knowledge base, certain areas of the curriculum have been minimized, leading to a notable reduction in urology education.^1^ A survey conducted in the United States revealed that, among 41 randomly selected accredited medical schools, only 52 % offered urology lectures or coursework before clinical rotations, with just 5 % (two schools) requiring a urology clinical clerkship.^2^ This decline in formal urology education contrasts sharply with the increasing prevalence of urological symptoms in the population.^3^

In Brazil, Medical Student Interest Groups (MSIGs), also known as Academic Leagues, provide an alternative way for students to complement their education. These MSIGs are student-led organizations affiliated with medical schools and designed to bridge the gap between theoretical knowledge and practical application.^4^ Each MSIG focuses on a specific medical field, guided by specialized faculty, and often includes direct patient contact. This structure allows students to gain experience in taking medical histories, performing physical examinations, and learning about common diseases in their area of interest. In some cases, students also perform simple, supervised procedures. Beyond clinical skills, MSIGs introduce students to the scientific community, encourage research activities, and provide early exposure to medical practice.^5^

The tradition of MSIGs is well established and widespread across Brazil's medical schools. There are actually 89 active MSIGs of different areas on our institution,^6^ and there are 37 Urological MSIGs enrolled on Brazilian Society of Urology.^7^ Notably, the first academic league in Brazil was founded in 1920 at our institution and remains active to this day.^8^

The Urological Medical Student Interest Group (UMSIG) was established in 2006 and has engaged hundreds of medical students since its inception. Admission to UMSIG requires participation in an annual introductory course and passing an admission test. While the course is open to all students, only third- and fourth-year students are eligible to take the exam. UMSIG activities occur weekly and include medical appointments for urological patients with low-complexity conditions, case discussions, observation of surgical procedures, urodynamic examinations, and classes on basic urological evaluation and prevalent conditions. Additionally, students are encouraged to participate in scientific research and attend urological conferences.

This study aims to assess the impact of UMSIG on its current and former members. Specifically, we evaluate their levels of satisfaction, overall experiences within the group, and the influence of participation on their decisions regarding future specialization.

## Materials and methods

This descriptive cross-sectional study was approved by the Research Ethics Committee of our institution (project number CAPPESQ 75,532,523.1.0000.0068), and informed consent was obtained from all participants. Data were collected via an electronic survey distributed by email to all former and current UMSIG members between December 22, 2023, and February 26, 2024. This manuscript was prepared in accordance with the STROBE statement for cross-sectional studies.

Out of 246 eligible participants (223 man and 23 women, comprising 230 former members and 16 current), contact information was obtained for 226 individuals (91.9 %), who were subsequently invited to participate. The invitation email included a link to a web-based survey containing 14 general questions applicable to all participants. Additionally, the survey featured tailored questions specific to the participants' current professional or academic status at the time of the survey: two questions for medical students, three for non-urologist physicians, and three for urologists or urology residents. For analysis, non-urologist physicians were further categorized into non-urologist surgeons and non-surgeon when relevant.

The survey collected data on age, gender, year of UMSIG entry, reasons for joining, evaluation of UMSIG activities, impact on specialty choice, current professional status, satisfaction levels, and perceived benefits.

The questionnaire was developed in Portuguese and tailored to the Brazilian medical education context. It consisted primarily of categorical and multiple-choice questions designed to assess demographic data, motivations for joining UMSIG, perceived educational value, and self-reported outcomes.

Given the modest overall sample size and subdivision into heterogeneous subgroups (e.g., medical students, non-urologist physicians, and urologists), together with the limited set of available covariates, we opted for a descriptive statistical approach. This methodology aligns with prior evaluations of Medical Student Interest Groups and was appropriate for the study’s objective of characterizing participant perceptions and experiences.

Survey responses were initially compiled using SurveyMonkey® software. Quantitative variables were summarized as medians with interquartile ranges, while qualitative variables were reported as absolute values, percentages, or proportions. Data analysis was conducted using GraphPad Prism version 10.0.0 (San Diego, CA, USA).

## Results

The survey yielded 111 responses, representing 49.1 % of invited members. Missing data were minimal, limited to a single incomplete response (< 1 % of participants). The median time to complete the questionnaire was 3 minutes and 45 seconds (IQR 2:35–4:54). The median age of participants was 31 years (27–35), and the majority were male (91.0 %). Response rates were similar for men (45.3 %) and women (43.5 %).

Participants were distributed across all years within the study period, with a median of six participants per year (IQR 3–10). Most respondents (80.2 %) had already completed medical school; 12.6 % were either practicing urologists or urology residents, and 3.6 % were in the process of applying for urology residency (Table 1).

**Table 1:** Academic and professional status of UMSIG survey respondents.

Professional status (*n* = 111)	n (%)
Other surgical specialist	24 (21.6)
Medical student	22 (19.8)
Non-surgical specialist	20 (18.1)
Urologist/Urology resident	14 (12.6)
Non-surgical resident	12 (10.8)
General practitioner^a^	8 (7.2)
Surgical resident	7 (6.3)
Urology residency applicant	4 (3.6)
Urology resident	2 (1.8)

a Never completed and not currently enrolled in any medical residency.

The primary motivations for joining UMSIG included a desire to learn more about urology (77.5 %), an interest in pursuing a surgical specialty (56.8 %), and recommendations from former participants (56.8 %). Other motivations were the intention to undertake a urology residency (43.2 %), the desire for practical experience treating urological patients (36.0 %), exposure to surgical procedures (33.3 %), and participation in scientific research (16.2 %) (Table 2).

**Table 2:** Reported motivations for participating in UMSIG.

Motivation (*n* = 111)	n (%)
To learn more about urology	86 (77.5)
To pursue a surgical residency (undecided at the time)	63 (56.8)
Based on recommendations from former participants	63 (56.8)
To undertake a urology residency	48 (43.2)
To gain practical experience treating urological patients	40 (36.0)
To gain exposure to surgical procedures	37 (33.3)
To participate in scientific research	18 (16.2)

Participation in scientific research during UMSIG varied: 67.6 % reported no engagement, while 32.4 % engaged in research projects, and 15.3 % completed at least one project.

Regarding involvement in other MSIGs, most respondents (69.4 %) participated in three or more, whereas 1.8 % had participated only in UMSIG.

The majority (91.8 %) deemed the frequency of UMSIG activities adequate. Most (71.2 %) reported that UMSIG complemented their curricular urology education; 26.1 % found it helpful but not essential, 2.7 % felt neutral, and none reported a negative impact.

Respondents identified unmet expectations, most commonly the desire for exposure to a greater diversity of urological conditions (56.8 %), and more opportunities to participate in surgical procedures (52.2 %). In line with these, the most frequent suggestion for improvement was to increase surgical participation opportunities (64.8 %), along with broader clinical exposure and greater research involvement (Table 3).

**Table 3:** Evaluation of UMSIG activities, unmet expectations, and suggestions for improvements.

Perception of UMSIG activities (*n* = 110)	n (%)
Adequate frequency	101 (91.8)
Insufficient (should have more)	8 (7.3)
Excessive (could be less frequent)	1 (0.9)

Impact on Urology knowledge (*n* = 111)	n (%)
Very positive: complemented learning beyond curriculum	79 (71.2)
Moderately positive: helpful but curriculum sufficient	29 (26.1)
Neutral	3 (2.7)
Negative	0 (0)

Unmet expectations (*n* = 111; Multiple answers allowed)	n (%)
Greater diversity of urological conditions	63 (56.8)
More opportunities in surgical procedures	58 (52.2)
Greater involvement in scientific research	25 (22.5)
More active patient care participation	3 (2.7)

Suggestions for improvements (*n* = 111; Multiple answers allowed)	n (%)
More surgical participation opportunities	72 (64.8)
Wider range of urological conditions	65 (58.6)
More research participation	37 (33.3)
More active patient care involvement	9 (8.1)
More theoretical classes	1 (0.9)

Among medical students, 50.0 % indicated that UMSIG participation reinforced their interest in urology and 40.9 % in the surgical field. Additionally, 36.3 % expressed a probable interest in pursuing urology as a future specialty, while others remained undecided or inclined toward other specialties.

Among non-urologist physicians, 48.0 % reported that UMSIG had no influence on their career choice, whereas 45.3 % said it reinforced their surgical inclination. Conversely, 78.6 % of urologists credited UMSIG with significantly influencing their decision to specialize in urology (Table 4).

**Table 4:** Influence of UMSIG participation on physicians' professional choices.

Group	Influence	n (%)
Non-urologists (*n* = 75)	No influence	36 (48.0)
	Reinforced inclination toward surgical field	34 (45.3)
	Reinforced inclination toward non-surgical field	5 (6.7)
Urologists (*n* = 14)	Significant influence: reinforced inclination toward urology	11 (78.6)
	Small influence: reinforced inclination toward urology	2 (14.3)
	No influence	1 (7.1)

Overall, UMSIG received a median rating of 9 (8–9) on a 0–10 scale (0 = worst, 10 = best). More than half (55.9 %) considered UMSIG to be superior to other MSIGs in which they had participated; 35.1 % rated it similar, 2.7 % inferior, 4.5 % declined to compare, and 1.8 % had no experience with other MSIGs. Finally, the likelihood of recommending UMSIG to other medical students was high, with a median score of 10 (8–10).

## Discussion

This study evaluated the long-term educational and professional outcomes of participation in the UMSIG since its inception in 2006. We found a high level of satisfaction, with participants reporting that UMSIG provided valuable learning experiences beyond the formal medical curriculum. Most respondents found the frequency of UMSIG activities to be appropriate for balancing academic responsibilities with extracurricular involvement, while offering consistent, structured exposure to urological practice. Most participants perceived UMSIG as having a positive impact on their urology education, with 71.2 % indicating that it complemented and enhanced their curricular learning by providing practical clinical experiences, case discussions, and access to surgical observation not otherwise available through standard coursework.

The participants self-reported that UMSIG influenced their career trajectories, reinforcing an in both urology and the broader surgical field for many respondents. However, the survey also identified areas for improvement, particularly the need for greater diversity of urological cases, expanded surgical participation opportunities, and more robust engagement in scientific research. These insights offer important guidance for the ongoing development of UMSIG and similar medical student interest groups.

Our study population consisted mainly of physicians, with 16 medical students. The study's high response rate (49.1 %) is notable, especially when compared to similar educational surveys that report response rates ranging from 2 % to 25 %.^9^, ^10^, ^11^, ^12^, ^13^, ^14^ This may reflect the concise and accessible nature of the survey, as well as respondents’ ongoing connection to UMSIG. Responses were predominantly from recent cohorts, likely due to a shorter interval since participation and easier contact. Minimal participation was observed from the 2020 cohort, likely attributable to the significant disruption of UMSIG activities during the COVID-19 pandemic.

MSIGs are increasingly recognized for complementing formal curricula,^4^ foster leadership skills,^15^ and promote early scientific engagement.^16^^,^^17^ Our findings reaffirm the educational value of MSIGs in supplementing deficiencies caused by the reduction of certain specialties, such as urology, in formal medical curricula.^1^^,^^2^ Specifically, UMSIG provided practical clinical exposure, research experience, and professional networking opportunities, all of which have been shown to influence career decision-making.

A desire to learn more about urology was the primary motivation for joining UMSIG (77.5 %), consistent with other studies of MSIG participation.^18^^,^^19^ Additionally, interest in a surgical career and recommendations from peers were significant motivating factors, each cited by 56.8 % of respondents. Notably, 43.8 % of students entering UMSIG expressed a specific interest in urology specialization. These findings are particularly relevant given the global decline in interest in surgical specialties, influenced by lifestyle considerations and work-life balance preferences.^20^, ^21^, ^22^, ^23^ In this context, structured extracurricular activities such as UMSIG serve a critical role in offering realistic exposure to specialty practice and facilitating mentorship, both of which are key in shaping specialty choice.^20^^,^^23^

The gender imbalance observed within UMSIG (91 % male respondents) reflects the broader underrepresentation of women in urology. Despite women comprising nearly 50 % of Brazilian physicians,^24^ only 2.6 % of urologists are women,^25^ compared to 10.1 % in the United States.^26^ While there is evidence of increasing female participation in urology residency programs globally,^26^ Brazil continues to have one of the lowest gender diversity rates among surgical specialties.^27^ UMSIG participation is open to all students, and recruitment is not subject to gender-based barriers. The response rate among female participants (43.5 %) was proportionally similar to that of male participants, suggesting comparable engagement. Although the number of female respondents was small, we did not identify trends suggesting different experiences. Promoting inclusive and supportive environments remains an important goal for surgical specialties historically underrepresented by women.

UMSIG participation had an enduring impact on former members. Among non-urologists, 52.0 % reported that UMSIG influenced their specialization choice, by either reinforcing an inclination toward surgery or clarifying a preference for non-surgical fields. Moreover, 87.8 % of non-urologists continue to apply knowledge gained from UMSIG in their current practice. Among urologists and residents, UMSIG was described as crucial in shaping career paths, with 78.6 % citing a significant influence on their choice to pursue urology, and 64.3 % indicating that UMSIG participation strengthened their curriculum and contributed to successful entry into urology residency programs.

Our findings also highlight the value of peer influence within MSIGs: a substantial proportion of members (56.8 %) reported joining UMSIG based on recommendations from previous participants. This dynamic likely reinforces the group’s high satisfaction ratings, including a median recommendation likelihood of 10 (IQR 8–10) and a median program rating of 9 (IQR 8–9), with over half of respondents considering UMSIG superior to other MSIGs at our institution.

This study has several limitations. First, it is based on retrospective, self-reported perceptions and is therefore susceptible to recall bias, particularly among older respondents. Second, the absence of a control group and of objective outcomes (e.g., match rates, academic metrics) limits the ability to establish causality. Third, although the response rate was relatively high for this type of alumni survey, the possibility of non-response bias cannot be excluded. Additionally, our modest sample size, heterogeneity across participant subgroups, and lack of detailed baseline variables limited the feasibility of conducting multivariate analyses. Finally, as a single-center study conducted in a specific Brazilian academic context, the generalizability of our findings may be limited. Future studies involving multiple institutions and prospective follow-up may help expand on our results.

## Conclusion

In conclusion, participants reported high levels of satisfaction with UMSIG and perceived it as a valuable complement to their formal medical education. They described positive educational experiences and reported that participation influenced their academic and professional interests. These findings are based on self-reported perceptions and should be interpreted as exploratory; future multicenter and prospective studies are warranted to further validate these findings. Nevertheless, they underscore the potential of structured extracurricular programs ‒ such as medical student interest groups ‒ to enhance exposure, mentorship, and engagement, especially in specialties with limited curricular presence.

## Declaration of generative AI and AI-assisted technologies in the writing process

During the preparation of this work, the authors used ChatGPT 4.0 in order to review grammar and improve readability. After using this tool/service, the authors reviewed and edited the content as needed and take full responsibility for the content of the publication.

## Authors’ contributions

Eduardo Zinoni Silva Pato: Methodology; writing-original draft; writing-review & editing.

Luiz Carlos Neves de Oliveira: Methodology; supervision.

Jose de Bessa Jr: Methodology; supervision; formal analysis.

Gabriel Kayano Freudenthal: Investigation; data Curation.

Guilherme Pimenta Roncete: Investigation; data Curation.

Luiza Rafih Abud: Investigation; data Curation.

Victor Hondo Silva de Moraes: Investigation; data Curation.

William Carlos Nahas: Supervision.

Cristiano Mendes Gomes: Conceptualization; methodology; supervision; project administration; writing-review & editing.

## Declaration of competing interest

The authors declare no conflicts of interest.
